# Mueller polarimetric imaging for fast macroscopic mapping of microscopic collagen matrix remodeling by smooth muscle cells

**DOI:** 10.1038/s41598-021-85164-y

**Published:** 2021-03-15

**Authors:** Olga Chashchina, Hachem Mezouar, Jérémy Vizet, Clothilde Raoux, Junha Park, Clara Ramón-Lozano, Marie-Claire Schanne-Klein, Abdul I. Barakat, Angelo Pierangelo

**Affiliations:** 1 Hydrodynamics Laboratory (CNRS UMR7646), Ecole Polytechnique, IP Paris, Paris, France; 2 LPICM (CNRS UMR 7647), Ecole Polytechnique, IP Paris, Paris, France; 3 LOB, CNRS, Inserm, Ecole Polytechnique, IP Paris, Paris, France

**Keywords:** Biophotonics, Imaging and sensing

## Abstract

Smooth muscle cells (SMCs) are critical players in cardiovascular disease development and undergo complex phenotype switching during disease progression. However, SMC phenotype is difficult to assess and track in co-culture studies. To determine the contractility of SMCs embedded within collagen hydrogels, we performed polarized light imaging and subsequent analysis based on Mueller matrices. Measurements were made both in the absence and presence of endothelial cells (ECs) in order to establish the impact of EC-SMC communication on SMC contractility. The results demonstrated that Mueller polarimetric imaging is indeed an appropriate tool for assessing SMC activity which significantly modifies the hydrogel retardance in the presence of ECs. These findings are consistent with the idea that EC-SMC communication promotes a more contractile SMC phenotype. More broadly, our findings suggest that Mueller polarimetry can be a useful tool for studies of spatial heterogeneities in hydrogel remodeling by SMCs.

## Introduction

The pathological complications of atherosclerosis, namely heart attacks and strokes, are the leading cause of mortality worldwide. Although chronic dysfunction of endothelial cells (ECs) which line the inner surface of blood vessels is a direct trigger for the development of atherosclerosis^1^, underlying smooth muscle cells (SMCs) are also critical players in the disease through their recruitment to the arterial intima and/or the deposition of extracellular matrix proteins to stabilize atherosclerotic plaques^1^. ECs and SMCs do not exert their physiological and pathological actions independently of one another. Indeed, there is mounting evidence that ECs and SMCs interact through various pathways, and this crosstalk has profound effects on the disease state^2–7^. Interestingly, while some of these interactions promote plaque growth, others appear to attenuate the size and the instability of atherosclerotic plaques^8^.

A key characteristic that SMCs exhibit is their remarkable plasticity in response to external stimuli such as vascular injury, inflammation, or lipoprotein accumulation. This plasticity manifests itself in the form of elaborate modifications of gene expression and associated phenotype switching^9–11^. In a mature healthy blood vessel, SMCs exhibit a “contractile” or differentiated phenotype characterized by the expression of markers specific to smooth muscle such as smooth muscle myosin heavy chain, smooth muscle *α*-actin, h-caldesmon and calponin, which are all important for the regulation of contraction. Upon injury, which is often related to EC layer damage, SMCs switch to a “synthetic phenotype” where they re-enter the cell cycle and exhibit an increased rate of proliferation, migration and synthesis of extracellular matrix components. This is accompanied by a decrease in the expression of smooth muscle-specific contractile markers. Studying SMC phenotype is an active field of research facing multiple challenges, partially due to SMC plasticity^12^. Many studies use in vivo animal models where the effect of different types of cells cannot be easily separated. Studying SMC interactions with ECs in vitro requires control of the initial experimental conditions. SMCs used for in vitro studies often have batch-specific and even cell passage-specific initial phenotype which can complicate the readout and reproducibility of co-culture studies^13^.

A potential solution to the problem of SMC phenotype tracking is to analyze it from a more functional perspective by focusing on the effect of SMCs on their substrate. Although both contractile and synthetic SMCs remodel the extracellular matrix, they are thought to do so in different manners. Contractile SMCs are presumed to remodel their matrix via contractions that alter the organization of the fibers within it. On the other hand, the impact of synthetic SMCs is exerted through their higher proliferation and migration rates as well as their increased rate of extracellular matrix protein deposition.

Recently Mueller polarimetric imaging has been reported as a promising tool to probe the microstructure of biological tissues, both ex vivo and in vivo^14–17^. In particular, it has been shown to be well suited for characterizing the collagen microstructure^18,19^, and effectively detect the modification of its organization in pre-cancerous and cancerous tissues^20–25^, or in radiofrequency-ablated lesions within myocardium^26^. The same technique has been demonstrated to also be capable of detecting structural changes in the wall of an obstructed bladder^27^. The great advantage of this technology is that it can be implemented with a large field of view (on the order of several square centimeters), while providing information on the microstructure of a sample, such as a biological tissue, by measuring its polarimetric properties without physical contact and without the use of chemicals.

The present study aimed to test, for the first time, the potential capability of Mueller polarimetric imaging for rapid and non-invasive tracking of interactions between SMCs and their extracellular matrix in vitro as well as to explore the effect of the SMC phenotype on these interactions. To this end, an original full-field Mueller polarimetric imaging system is used to obtain a macroscopic mapping of the local microscopic organization on a scale of approximately 100 µm of a collagen hydrogel with embedded SMCs either in the presence or absence of ECs on its surface to modulate SMC phenotype. The promising results of this study pave the way for using Mueller polarimetric imaging as an innovative approach to accurately characterize, with a wide field of view and in only a few seconds, changes in the microstructural organization of collagen hydrogels under different conditions. We expect that these results, in turn, would enhance SMC-EC co-culture research and open avenues for extending the proposed approach to artificial or engineered arteries.

## Results

SMCs embedded in a collagen hydrogel matrix can remodel their environment through both protein deposition and contraction^28^. Nevertheless, this remodeling is dependent on SMC phenotype and activity. In laboratory setting, the phenotypic state of the cells can vary among samples, leading to complex interpretation of the experimental results of co-culture studies. In this work, we tested the feasibility of a new method, Mueller polarimetric imaging, for detecting SMC activity in an in vitro design by analyzing the effect of these cells on collagen hydrogel within which they are embedded.

**Figure 1:**
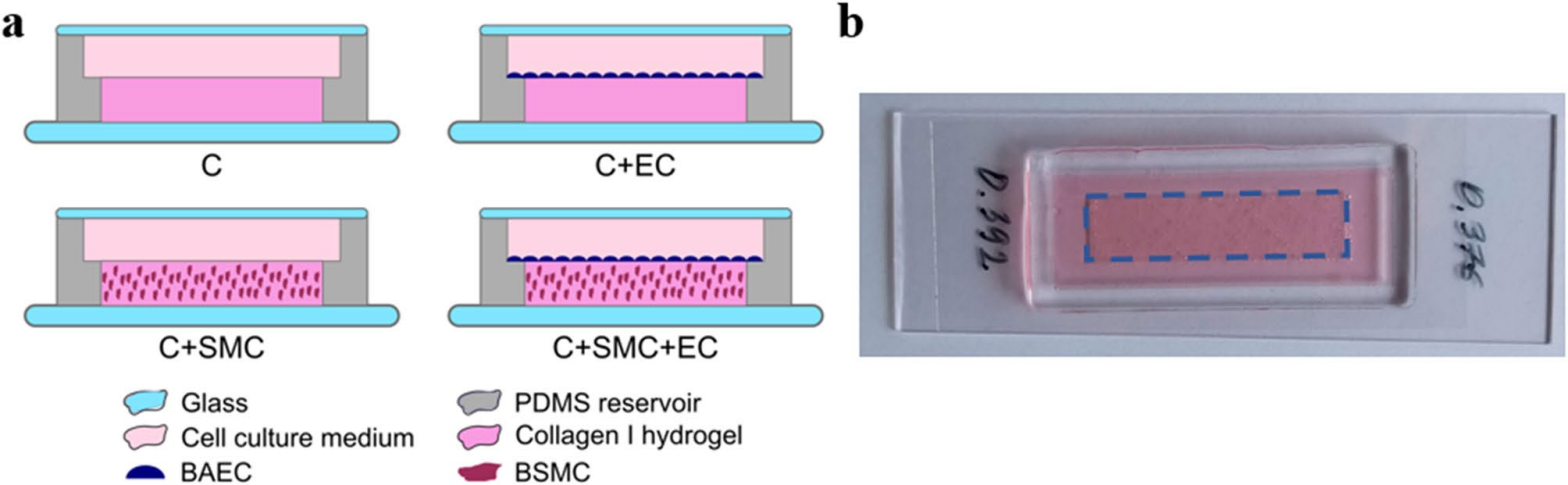
(**a**) Schematic drawing of the four types of analyzed samples: collagen I hydrogel alone (C); (i) collagen I hydrogel covered with ECs (C + EC); (ii) collagen I hydrogel containing SMCs (C + SMC); (iii) collagen I hydrogel containing SMCs and covered with ECs (C + SMC + EC). (**b**) Photograph of a typical sample after preparation. The central rectangle (0.75 cm × 3 cm) corresponds to the collagen hydrogel location after casting. BAEC: bovine aortic ECs; BSMC: bovine SMCs.

As shown in Fig. 1, four types of samples were considered for the analysis: (i) collagen I hydrogel alone (C); (ii) collagen I hydrogel covered with ECs (C + EC); (iii) collagen I hydrogel containing SMCs (C + SMC); and (iv) collagen I hydrogel containing SMCs and covered with ECs (C + SMC + EC). All samples were prepared in a two-level polydimethylsiloxane (PDMS) reservoir plasma bonded to a glass slide. A collagen I hydrogel with or without SMCs was poured into the bottom level of the reservoir and left to polymerize for 20 min at 37 °C. The hydrogel was then covered with ECs for some samples and with cell culture medium. Each sample was then cultured for 3 days prior to polarimetric measurements.

Based on previous work^9–11^, significantly higher contractility is expected when SMCs are co-cultured with ECs, which would functionally reflect a more contractile phenotype.

### Assessment of sample functional characteristics

**Figure 2:**
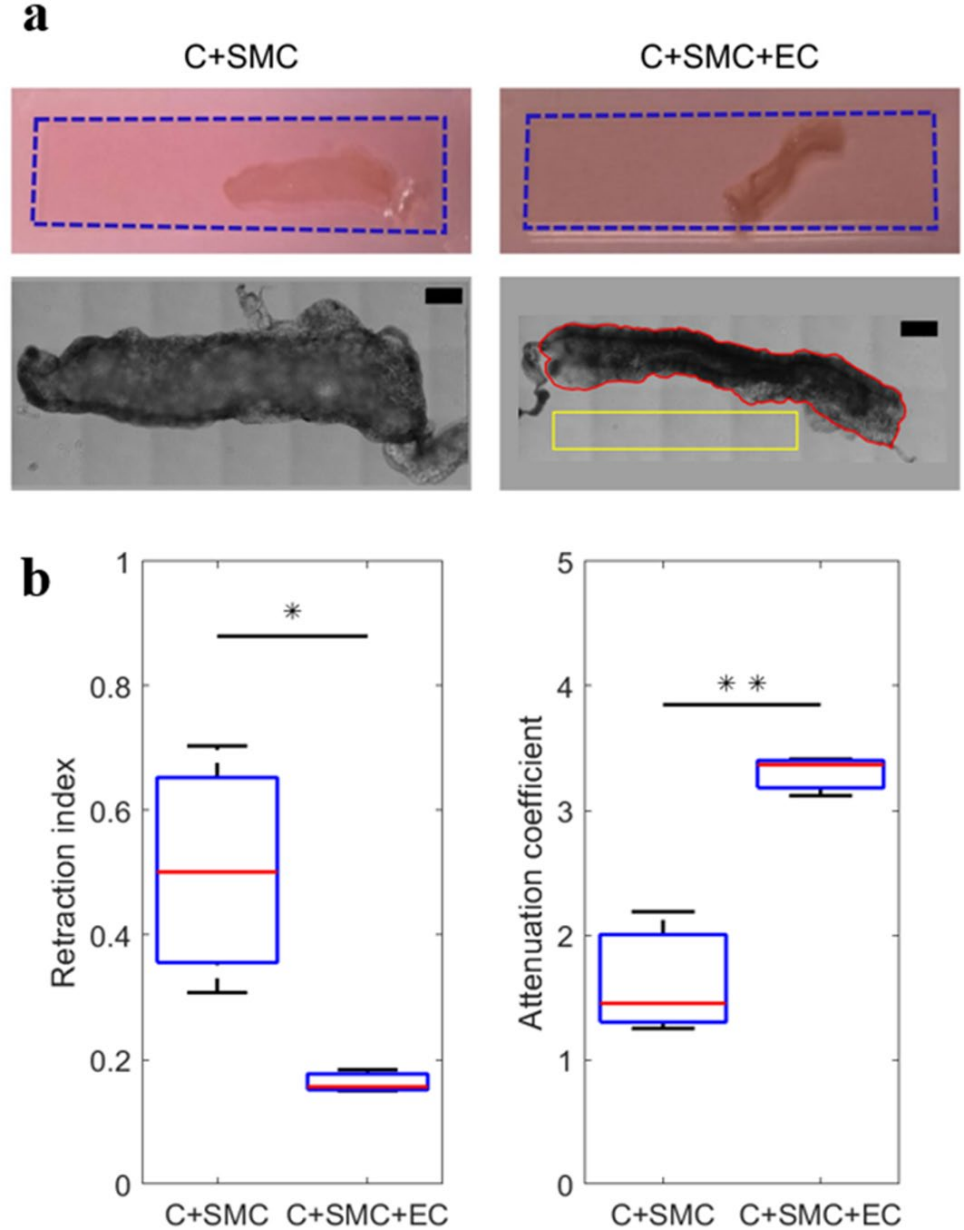
(**a**) Examples of contracted C + SMC and C + SMC + EC samples. Top row: representative photographs after 3 days of culture. The dashed blue rectangle represents the initial contour of the sample at day 0. Bottom row: zoomed bright-field images of the same samples. Scale bar corresponds to 1.5 mm. As an example, the area occupied by the considered C + SMC + EC contracted sample (projected on the image plane) at day 3, is delimited by the continuous red line. The retraction index is obtained by calculating the ratio between the surface delimited by the continuous red line and that occupied by the sample without contraction at day 0 (delimited by the dashed blue rectangle). The attenuation coefficient is obtained by calculating the ratio between the average transmitted light intensity in the area inside the continuous red line and that of the background inside the continuous yellow rectangle. (**b**) Statistical comparison of the retraction index and the attenuation coefficient for C + SMC and C + SMC + EC samples. Three samples were analyzed for each considered condition. In the box plots, the red lines represent the median values, while the whiskers extend to the most extreme data points. *p < 0.050. ** *p* < 0.010.

SMCs elongate within the collagen matrix, while ECs form a confluent monolayer on the top surface of the collagen hydrogel as shown in Fig. S1. For the SMC density under consideration (10^6^ cells/ml), SMC contraction is not sufficient to detach the collagen from the PDMS chamber over 3 days of culturing. Therefore, for the gel contractility assay, the gel was manually detached from the PDMS chamber 1 h after preparation. The C + SMC and C + SMC + EC samples were then left to contract for 3 days while free of external constraints as shown in Fig. 2a. After bright-field imaging, the contractility of C + SMC and C + SMC + EC samples was assessed and compared by measuring two parameters: (i) the retraction index, defined as the ratio between the sample’s surface area projected on the image plane at day 3 and the surface area occupied by the same non-contracted sample at day 0; (ii) the attenuation coefficient, defined as the ratio between the average transmitted light intensity measured at day 3 at the location of the contracted sample and that measured in the area around the sample, considered as the baseline. As shown in Fig. 2b, the retraction index was 0.163 ± 0.015 (Standard deviation (S.D.)) for the C + SMC + EC samples vs. 0.504 ± 0.162 (S.D.) for the C + SMC samples. Similarly, the mean attenuation coefficient for the C + SMC + EC samples was twice larger than that for the C + SMC samples. Thus, the C + SMC + EC samples were observed to be significantly more contracted (*p* < 0.050) and optically dense (*p* < 0.010) at day 3 relative to the C + SMC samples. These results suggest that, after 3 days of culture, the SMCs exhibit a strong contractile phenotype and induce much higher modification of their substrate in the presence of ECs. A certain degree of contractility was indeed observed for the samples containing SMCs alone (i.e. no ECs); however, this contractility appeared to be much lower than the case of the samples containing both SMCs and ECs. Moreover, the higher value of the standard deviation obtained for the C + SMC samples, both for the retraction index and the attenuation coefficient, suggests significant variability in the contractility of SMCs from one sample to another. On the other hand, a much smaller standard deviation was obtained for the C + SMC + EC samples, for both considered parameters. This can be attributed to the fact that, due to the strong contractility of SMCs in the presence of ECs, each analyzed sample for this category attained its full compaction limit.

### Polarimetric measurements

By contracting, SMCs can modify the microscopic structure of the surrounding collagen hydrogel in which they are embedded. This can be unambiguously detected if the collagen fibers in the initial hydrogel without any cell do not have a preferred orientation. Such a condition has been verified in our hydrogels using polarization-resolved second harmonic microscopy (pSHG). The pSHG images confirm the presence of thin collagen fibers interlaced with no preferred orientations^29^ (see Materials and Methods and Fig. S2). Starting from this isotropic hydrogel, SMC contraction may induce a level of local fiber re-organization and alignment. This modification in the microscopic structure of the collagen hydrogel can produce changes in its polarimetric properties, which are detectable at a macroscopic scale. The main challenge of this study is to show that the measurement and the quantification of the polarimetric properties of the collagen hydrogel can provide information about the phenotype of SMCs embedded within it. For this purpose, an original full-field Mueller Polarimetric Imager (MPI) in reflection configuration (Fig. S3) with a resolution of ~ 100 µm/pixel was used to analyze several samples of collagen hydrogel under the different conditions previously described in Fig. 1a. In particular, the polarimetric response of the C + SMC and C + SMC + EC samples was compared in order to establish if the MPI can optically detect changes in contractility of SMCs induced by the presence of ECs. As a control, C samples were analyzed to determine the polarimetric response of the collagen hydrogel alone. Furthermore, C + EC samples were analyzed to determine if ECs alone can induce a detectable modification in the structure of the collagen hydrogel. For these experiments, collagen hydrogel samples were not detached from the PDMS chamber. In this way they remained flat and non-contracted during the imaging process. More specifically, the MPI allowed determination of the Mueller matrix **M**_S_ for each sample at different wavelengths. **M**_S_ consists of a 4 × 4 real-component matrix providing the comprehensive polarimetric characterization of the explored sample. Subsequently, the Lu-Chipman decomposition was used to extract the main polarimetric properties of the analyzed sample from **M**_S_^30^, specifically the total depolarization ($\Delta$) and the linear retardance (R). The parameter $\Delta$ reflects the sample's ability to depolarize both linear and circular polarization states. It ranges from 0 for a non-depolarizing sample to 1 for a purely depolarizing sample. The parameter R quantifies the difference in optical phase shifts between two principal polarization directions for light backscattered by the sample. It generally varies between 0° and 180° for biological tissues. More details concerning the experimental setup and the data processing by Lu-Chipman decomposition are provided in the Materials and Methods section.

**Figure 3:**
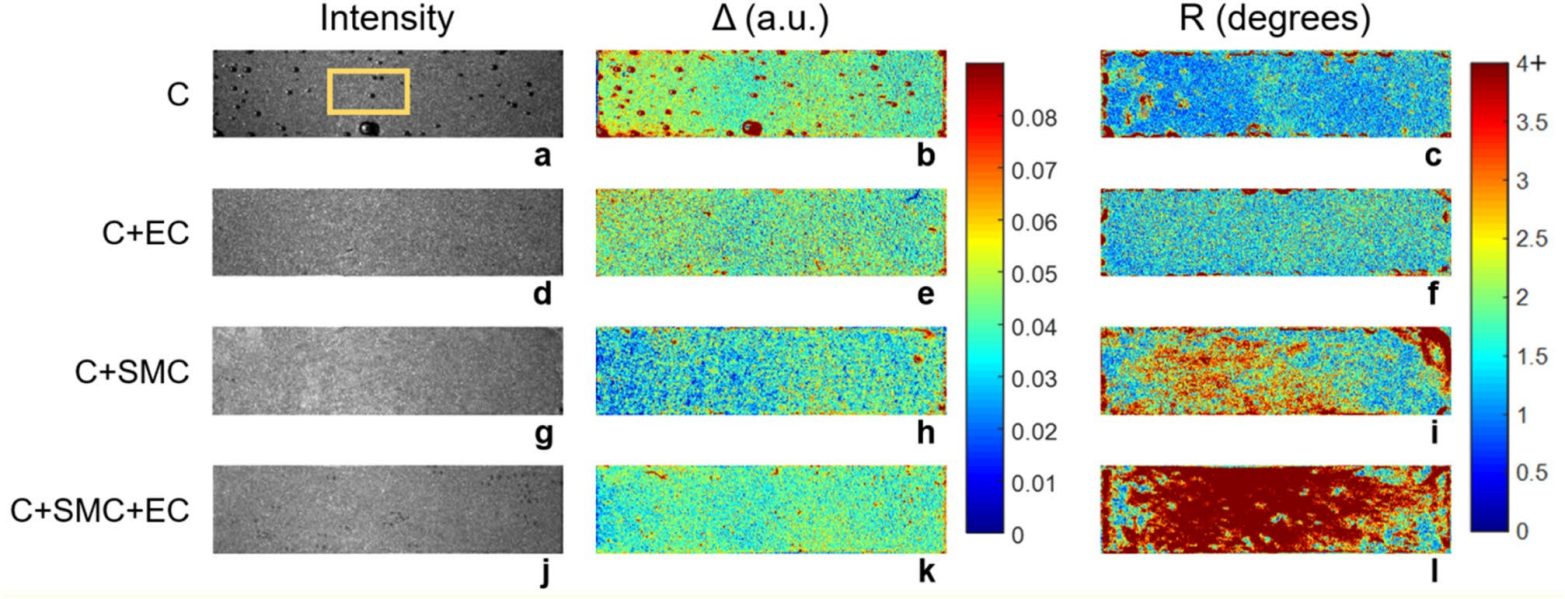
Intensity and polarimetric parameters of representative samples for each considered condition: (**a–c**) collagen hydrogel alone (C, sample 1), (**d–f**) collagen hydrogel with ECs on its top surface (E + EC, sample 1), (**g–i**) collagen hydrogel with embedded SMCs (C + SMC, sample 2), and (**j–l**) collagen hydrogel with embedded SMCs and ECs on its top surface (C + SMC + EC, sample 2). The first column shows the monochromatic unpolarized intensity image (arbitrary units), corresponding to the non-normalized m_11_ coefficient of the measured Mueller matrix **M**_S_. The second and third columns show the parameters Δ and R, respectively. The dimensions of the imaged samples are 0.75 cm × 3 cm.

In Fig. 3, each row depicts the parameters $\Delta$ and R for a representative sample from each experimental category. The first column shows the monochromatic unpolarized intensity image, corresponding to the non-normalized m_11_ coefficient of the measured Mueller matrix **M**_S_. The second and third columns show the parameters Δ and R, respectively. No difference is observed in the m_11_ coefficient among the four experimental conditions. However, polarimetric images appear to enable improved contrast compared to conventional unpolarized intensity images. An initial qualitative inspection of the obtained results suggests that the parameter R appears to be very promising for detecting contractility. Indeed, this parameter increases slightly for the C + SMC samples and strongly for the C + SMC + EC compared to the two control groups. Conversely, $\Delta$ appears to be less pertinent to optically detect contractility. Moreover, all analyzed samples were observed to have a highly spatially heterogeneous polarimetric response for both Δ and R, even if they appeared to be homogeneous in conventional unpolarized intensity images. For each sample, the macroscopically observable spatial heterogeneity in measured polarimetric parameters reflects the modification of its microstructure produced during the different preparation steps, specifically by the imperfect mixing of the collagen solution, its casting, and flattening. The last two processes have particular effect on the sample’s extremities. For this reason, to avoid this preparation bias, only the area at the center of each sample and far from the edges (denoted by the yellow rectangle in Fig. 3a) was considered for a more quantitative analysis. The mean value of each polarimetric parameter was calculated for all pixels contained in the selected zone. This approach was used to compare the Δ and R parameters among the different experimental conditions.

**Figure 4:**
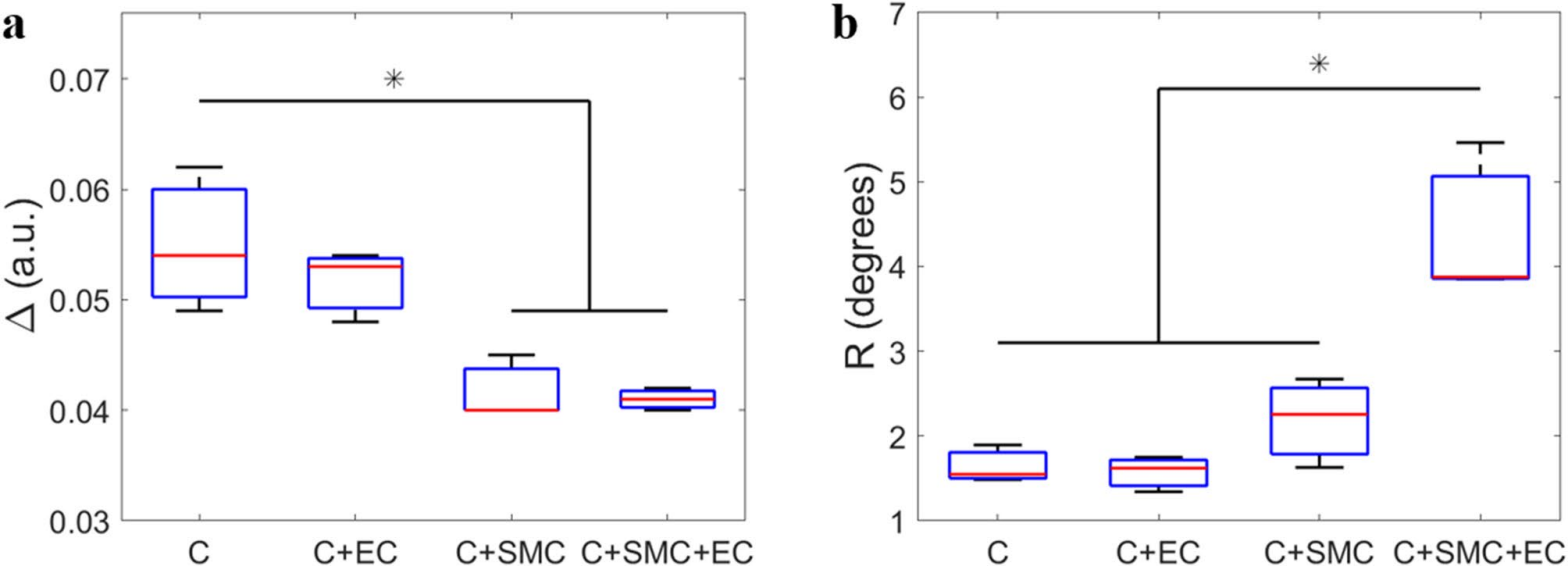
(**a**) Depolarization and (**b**) retardance for collagen hydrogel alone (C), collagen hydrogel with ECs on its top surface (E + EC), collagen hydrogel with embedded SMCs (C + SMC), and collagen hydrogel with embedded SMCs and ECs on its top surface. The mean value of each polarimetric parameter per sample was calculated for all pixels contained in the area shown by yellow rectangle in Fig. 3a. The red lines in the box plots denote the median values among samples means, whereas the whiskers extend to the most extreme data points. *p < 0.050. Three independent samples were analyzed for each condition.

Statistical analysis of depolarization showed that the C + SMC and C + SMC + EC exhibit smaller depolarization values than the C and C + EC samples globally (Fig. 4). The observed difference was significant (*p* < 0.050) with the exception of the pair-wise comparison between C + EC and C + SMC (*p* = 0.058) that we attributed to limited statistics. The presence of SMCs in the volume of the collagen hydrogel should increase the percentage of the light that is forward scattered. This is probably due to the size of SMCs (on the order of several tens of microns) which is considerably larger than the wavelength of the light (550 nm) used to perform the measurements^31^. This process increases the path of a certain percentage of photons inside the sample before being collected by the detector, thus increasing the probability that they are absorbed during their propagation. Due to the increase in photon forward scattering, the presence of SMCs in the collagen hydrogel also globally produces an increase in the absorption of light by the sample with a consequent decrease in measured depolarization. No significant difference (*p* = 0.737) was observed in $\Delta$ between the C and C + EC samples, suggesting that the presence of ECs on the surface of the collagen hydrogel does not alter its scattering bulk properties. Moreover, no significant difference (*p* = 0.997) was observed in $\Delta$ between the C + SMC and C + SMC + EC samples, suggesting that this parameter, taken alone, is not able to detect the change in SMC contractility induced by the presence of ECs. However, it can provide useful information about the increased cellular density within the bulk of the sample, which we intend to investigate in a separate study.

Statistical analysis of retardance, on the other hand, showed that the C + SMC + EC samples are characterized by a value of R that is significantly greater (*p* < 0.050) than the C + SMC, C + EC and C samples. Moreover, while R is increased for the C + SMC samples relative to the C + EC and C samples, this increase did not attain statistical significance (*p* > 0.550). These findings suggest that R may provide an indication of the switch of SMC phenotype from synthetic to contractile (or at least of the functionally observed augmented contraction) upon having ECs present on the surface of the collagen hydrogel. More generally, a non-negligible value of R, measured at the macroscopic scale in biological tissues, can be attributed to the anisotropy of the extracellular matrix, due to a certain level of microscopic organization of the fiber structures^32^. In this study, the increasing value of R for the C + SMC + EC samples compared to the C + SMC, C + EC and C samples is considered to reflect a situation of more contractile SMCs that actively increase the local alignment of fibers that constitute the extracellular matrix (the collagen hydrogel). Moreover, the larger value of R (even if not statistically significant) for the C + SMC samples relative to the C + EC and C samples is consistent with the fact that SMC state between the synthetic and contractile phenotypes is not perfectly binary^10^. Therefore, even without ECs on the collagen hydrogel surface, SMCs can exhibit moderate contractile activity. The results described in this section are globally in good agreement with those obtained for the gel contractility assay described in the previous section. In polarimetric experiments, unlike the mechanical studies, a larger standard deviation was observed for the samples belonging to the C + SMC + EC category, as shown in Fig. 4b. This highlights the variability in the local contraction force from one sample to another, which can be attributed to several concomitant factors such as intrinsic contractility differences among SMCs as well as variations in the local cell and collagen density.

#### Endothelial cell effect on polarimetric parameters

Both Δ and R were very similar for the C and C + EC samples, suggesting that ECs at the top gel surface do not modify the bulk of the gel substrate sufficiently to be detected by MPI. It should be noted here that this does not exclude the possibility of some local remodeling induced by the ECs near the gel surface as a result of the expected deposition of basement membrane proteins by the ECs^33,34^. However, this is not the central topic of this study and will be part of future investigations. It should also be noted that the C samples showed a larger standard deviation for Δ than the other samples. This effect is primarily attributed to the presence of small air bubbles encapsulated in some C samples which led to spots of higher Δ (Fig. 3a,b)**.**

#### Sample heterogeneity assessment

**Figure 5:**
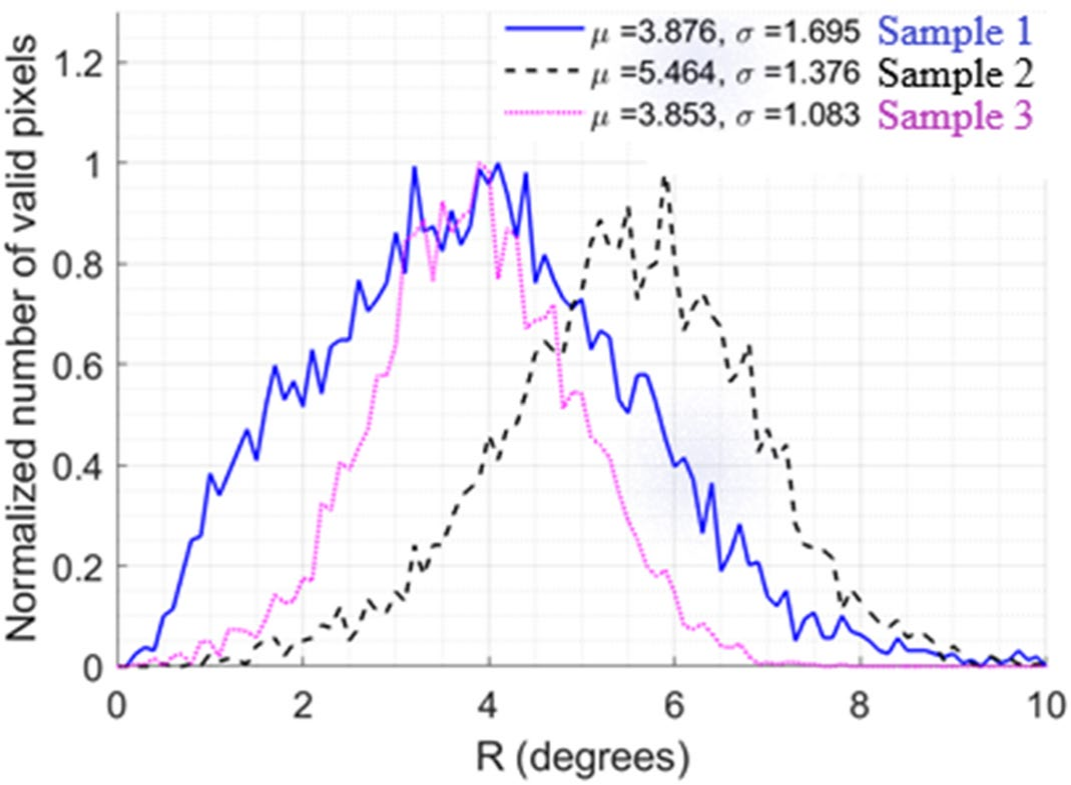
Retardance histograms for the analyzed C + SMC + EC samples based on the measurements within the yellow zone shown in Fig. 3a. *µ* corresponds to the mean retardance value of an individual sample. *σ* corresponds to the standard deviation of the mean.

**Figure 6:**
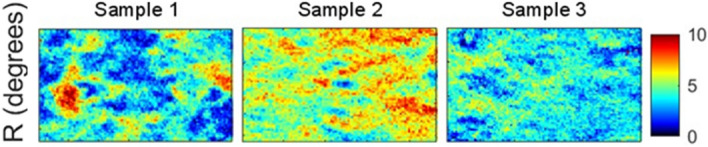
Retardance map for the analyzed areas of three C + SMC + EC samples.

In order to evaluate the spatial homogeneity of a sample, the histograms of Δ and R were calculated for all pixels contained within the rectangular window defined in its center (far from the edges). An example of such a window is shown in Fig. 3a. The histograms of the parameters Δ and R were traced for all the analyzed samples, as shown in Fig. S5 and Fig. S6 for Δ and for R, respectively (supplementary information). Larger spatial variability was observed for R, especially for the C + SMC + EC samples. Only the results obtained for this specific case are shown and discussed in detail here. For each histogram, the average value µ was computed as well as its standard deviation σ, which is a measure of the spatial heterogeneity of the sample for the selected zone. As shown in Fig. 5, the value of µ is very similar for samples 1 and 3. However the value of σ is 60% larger for sample 1 than for sample 3. Moreover, the value of µ is higher for sample 2 compared to the other samples. Otherwise, the value of σ for sample 2 is intermediate between those of samples 1 and 3. The image of retardance for these samples, given in Fig. 6, clearly corroborates the observed differences for the obtained histograms. Sample 1, for example, appears to be characterized by islands and valleys with higher and lower values of R, while sample 3 exhibits a much more spatially homogeneous distribution. This can explain why while the value of µ is similar for both, sample 1 is characterized by a greater value of σ than sample 3. Both heterogeneities in SMC density distribution and variations in local collagen density could be sources for this heterogeneity: a locally elevated cell density would lead to local variations in contractility, while differences in collagen density may lead to local variations in collagen deformations for the same applied contractile stress.

## Discussion

Although SMC switching between the contractile and synthetic phenotypes plays an important role in normal vascular function and in the development of various diseases including atherosclerosis and arterial restenosis, our understanding of this process remains far from complete. More generally, minimally invasive tools that predict the state of contractility of SMCs within a macroscopic fibrous structure, characterize the impact of SMC contractility on the surrounding extracellular matrix, and provide an assessment of the spatial homogeneity of the sample and its contractility state are useful both for fundamental research as well as for bioengineering applications in the cardiovascular field including the engineering of vascular grafts.

The present study demonstrated that MPI represents a very promising technique for addressing these issues. Indeed, this imaging modality enables the acquisition, within only a few seconds, of a macroscopic map of the tissue polarimetry properties, which are linked to its composition and microscopic organization.

The proposed approach enables assessing the modification of a collagen hydrogel by SMCs. More specifically, we were able to show that the retardance signal was consistent with enhanced contractility and hence a more “contractile” SMC phenotype upon co-culture with ECs and with a more “synthetic” phenotype when ECs were absent. The results also suggest that SMCs can significantly modify their environment after only 3 days of culture. In a future study, we aim to correlate Mueller polarimetric analysis with SHG imaging to visualize and quantify the microscopic collagen reorganization by SMCs which generates the observed increase in tissue anisotropy.

In addition, the Mueller polarimetry approach allowed us to rapidly analyze sample-to-sample heterogeneity in the contractility response as well as to assess spatial inhomogeneities within the sample arising possibly from preparation methods. These capabilities are quite interesting and are not readily available via conventional optical means. Thus, Mueller polarimetry promises to be a useful tool for minimally invasive structural quality assessment of sensitive constructs such as implantable grafts and for the optimization of sample manufacturing.

An additional benefit of applying this novel Mueller polarimetry approach to a controlled in vitro system is that it helps interpret the physiological relevance of the polarimetric parameters in complex biological tissues. The proposed approach is a crucial step for determining the relation between macroscopically measured polarimetric parameters of a tissue and its physiological properties. This study paves the way to new strategies aimed at investigating the biological properties of tissues both ex vivo and in vivo.

## Materials and methods

### Cell culture

Bovine aortic endothelial cells (ECs; Cell Applications, San Diego, USA) and bovine aortic smooth muscle cells (SMCs, Cell Applications) were cultured in corresponding cell growth medium (Cell Applications) at 37 °C in a 5% CO_2_ incubator. Cells were detached from the adhering surface for passage or experiments using TrypLE (Life Technologies, Carlsbad, USA). ECs were used between passages 4 and 8 and SMCs between passages 14 and 18.

### Collagen extraction

Rat tail tendons were dissolved in a solution of 0.01 mol/L hydrochloric acid (Sigma-Aldrich, St. Louis, USA). The solution was then centrifuged at 30,000 g, 4 °C for 1 h. The supernatant was cooled to − 80 °C, lyophilized and stored at − 20 °C for up to 6 months. For experiments, the lyophilized collagen was reconstituted to 12 mg/mL in 0.01 mol/L HCl solution and stored at 4 °C. Rat tails were kindly provided by Dr. Diana Zala (IPNP, UMRS 894). No live animals were handled in the current study.

### Experimental sample assembly

A custom-made polydimethylsiloxane (PDMS, Sylgard, Sarasota, USA) reservoir with a double well structure was plasma-bonded to a microscope slide and sterilized with ultraviolet (UV) light for 20 min. A collagen hydrogel solution (1 mL of 12 mg/mL collagen in 0.01 mol/L HCl, 200 µL 10X concentrated Dulbecco’s Modified Eagle’s Medium (DMEM; Sigma-Aldrich), 756 µL EC culture medium (Cell Applications), 43.5 µL of 1 mol/L NaOH (Sigma-Aldrich) for a final collagen concentration of 6 mg/mL) containing 10^6^ SMCs/ml for the C + SMC and C + SMC + EC samples, was pipetted into the reservoir, covered with an additional flat PDMS plate to flatten the collagen surface and left to polymerize for 20 min at 37 °C. The total gel layer thickness was 378 ± 40 (S.D.) µm measured with a microscope with a moving in z-direction stage. After the PDMS plate removal, 75 × 10^4^ ECs were seeded on top of the gel for the C + EC and C + SMC + EC samples, covered with DMEM (Gibco) supplemented with 10% fetal bovine serum (Gibco) and 1% penicillin (Gibco), and incubated for 3 days with daily medium changes. This seeding density led to a confluent monolayer on the sample’s surface within 24 h. Prior to polarimetric measurements, the system was sealed with a coverslip via PDMS-glass adhesion and capillary forces between the cell medium and the glass slide.

### Gel contraction assay

For the gel contraction assay, the samples were prepared in the same way as described above with the exception that the collagen hydrogel was detached from the PDMS reservoir with a spatula 1 h after ECs seeding. Bright-field images of these samples were acquired using an inverted microscope (Nikon Eclipse Ti-U, Japan), equipped with a charge-coupled device (CCD) camera (Orca AG ; Hamamatsu, Japan) with a 4 × objective (Nikon) and a motorized x–y stage. Image analysis (selected area and intensity measurements) was performed using FIJI, an ImageJ suite^35^.

### Acquisition and analysis of polarimetric images

Polarimetric images of each sample were acquired using a custom-made Mueller Polarimetric Imager (MPI) built in-house (Fig. S3). A 150-W halogen source (Olympus CLH-CS) generating incoherent white light is used to illuminate the sample of interest. It is coupled with a photodiode installed near the source to monitor the temporal stability of the emitted light power. The polarization of the light beam impinging on the sample is modulated by a Polarization State Generator (PSG) which allows consecutive production of four independent probing polarization states, described by four Stokes vectors S_Wi_ (i = 1 to 4), regrouped together as columns of the modulation matrix **W**. Each of the four polarization states produced by the PSG, after interacting with the sample, is analyzed through four independent consecutive polarization configurations of a Polarization State Analyzer (PSA) placed in front of a monochromatic CCD camera (f080b Allied Vision, 512 × 386 pixels in binning mode). These four polarization configurations of the PSA are described by four Stokes vectors S_Ai_ (i = 1 to 4), grouped together as rows of the analysis matrix **A**. The PSG and the PSA of the MPI are based on ferroelectric liquid crystals, whose construction and optimization are specified in^36^. Total of 16 independent intensity measurements form a 16-component intensity matrix:

1 $${\mathbf{B}}_{{\text{S}}} = {\mathbf{A}}\cdot{\mathbf{M}}_{{\text{S}}} \cdot{\mathbf{W}},$$

where **M**_S_ is the Mueller matrix of the explored sample. **M**_S_ can be obtained from Eq. 1 as:

$${\mathbf{M}}_{{\text{S}}} = {\mathbf{A}}^{{ - {1}}} \cdot{\mathbf{B}}_{{\text{S}}} \cdot{\mathbf{W}}^{{ - {1}}} ,$$

assuming the **W** and **A** matrices have been previously accurately determined through a calibration procedure. In particular, the Eigenvalue Calibration Method (ECM) is used to calibrate the MPI^37^. The calibration is performed by placing a sandblasted metallic plate (SMP) between the PSG and the PSA. It corresponds to a polarimetrically neutral reflecting system characterized by a Mueller matrix **M**_P_ ~ **I** where **I** is the identity matrix. The **W** and **A** matrices are determined for each pixel of the image from measurements performed on reference optical samples with well-known polarimetric properties^38^, consecutively inserted between the PSG and the SMP (position indicated as C1 in Fig. S3b).

A zoom module placed in front of the CCD camera allows modifying the size of the image on the detector from 3 mm up to 5 cm. A wheel containing several dichroic filters is placed in front of the CCD camera enabling measurements with wavelengths between 450 and 700 nm (steps of 50 nm, bandwidth of 40 nm).

In this work, a field of view of 32 × 24 mm^2^ was chosen, corresponding to a nominal resolution of 62 µm/pixel. Practical resolution was ~ 100 µm/pixel. Samples were positioned on the SMP which played the role of a reflector as the collagen hydrogel appeared to be optically transparent. Measurements were performed for each sample at 550 and 650 nm. However, no significant differences were observed between the results obtained for these two wavelengths due to the absence of blood in the analyzed samples. For this reason, only results at 550 nm are shown.

The Mueller matrix **M**_S_ obtained for each sample was interpreted using the polar decomposition proposed by Lu and Chipman^30^. This decomposition describes **M**_S_ as the product of three matrices:

$${\mathbf{M}}_{{\text{S}}} = {\mathbf{M}}_{\Delta } {\mathbf{M}}_{{\text{R}}} {\mathbf{M}}_{{\text{D}}}$$

where **M**_∆_ is the Mueller matrix of a depolarizer, **M**_R_ of a birefringent medium and **M**_D_ of a diattenuator. The most relevant polarimetric parameters characterizing our samples were the depolarization ($\Delta$) and the linear retardance (R) which can be expressed as:

$$\Delta =1-\frac{\left|\mathrm{a}\right|+\left|\mathrm{b}\right|+|\mathrm{c}|}{3}$$

where a, b and c are the eigenvalues of **M**_∆_.

$$\mathrm{R}=\mathrm{arccos}\left(\frac{\mathrm{tr}({\mathbf{M}}_{\mathrm{R}})}{2}-1\right)$$

where $\mathrm{tr}({\mathbf{M}}_{\mathrm{R}})$ is the trace of ${\mathbf{M}}_{\mathrm{R}}$. The quantitative analyses of polarimetric properties obtained for each sample were performed using a proprietary MatLab routine developed in-house.

As the PSG and the PSA are sensitive to temperature fluctuations, the MPI was recalibrated every hour to adjust for any parameter drift. In addition to the calibration, we repeatedly measured the **M**_P_ and corresponding $\Delta$ and R of the SMP alone. Since the SMP can be considered with a good approximation as polarimetrically neutral, this procedure established the instrument error to be ~ 0.002 ± 0.002 (S.D) a.u. for ∆ and ~ 0.424° ± 0.19° (S.D) for R (Fig. S4). For all analyzed samples, the measured ∆ and R significantly exceeded the noise level.

### Acquisition and analysis of Second Harmonic Generation (SHG) images

SHG images were acquired using a custom-built laser-scanning multiphoton microscope. Excitation was provided by a femtosecond titanium − sapphire laser (Mai-Tai, Spectra-Physics) tuned to 860 nm, scanned in the XY directions using galvanometric mirrors and focused using a 25 × objective lens (XLPLN25XWMP2, Olympus), with lateral (resp. axial) resolution of typically 0.4 μm (resp. 1.2 μm). A set of linear polarizations with different orientations was used to perform polarization-resolved measurements (pSHG). All pSHG images were acquired at 18 excitation angles regularly spaced between 0 and 180°, using 100 kHz acquisition rate and 170 × 170 nm^2^ pixel size. Power at the sample was typically 35 mW and images were collected using trans-detection. After applying 2 × 2 binning, pSHG images were processed as already described^39^ to provide two images: (i) the average of all images acquired with the set of linear polarizations to mitigate any effect of the excitation polarization orientation; and (ii) a map of the collagen main orientation within the image plane in every voxel. The latter pSHG image is displayed using the HSV look-up-table, where H is the orientation displayed in the inset and V is the brightness, which is set to 1 if the pSHG processing is satisfactory (R^2^ > 0.5) or to 0 otherwise. Four different acellular samples (C) were observed to assess the sample structure.

To characterize the distribution of the collagen orientation in each sample, two parameters are calculated: (i) the normalized entropy which reflects the uniformity of the distribution:

$$S=-\frac{1}{\mathrm{ln}(180)}\sum_{\theta }{p}_{\theta }\;\mathrm{ln}\;{p}_{\theta }$$

and (ii) the circular variance which indicates how the distribution is organized around the mean orientation:

$$CV=1-\left|\sum_{\theta }{p}_{\theta } {e}^{2i\theta }\right|$$

where ${p}_{\theta }$ is the proportion of pixels that have an orientation equal to $\theta$. These two parameters vary from 0 for a distribution with a single orientation to 1 for a distribution with no preferred orientation.

### Statistical analysis

For each experimental set-up and each considered condition, 3 independent samples were tested and compared. All statistical comparisons among several data series were computed using a one-way ANOVA followed by Tukey’s post hoc test. A value of *p* < 0.05 for the p-test was considered statistically significant.

## Supplementary Information

Supplementary Information
