# Supplementary material for: Mueller polarimetric imaging for fast macroscopic mapping of microscopic collagen matrix remodeling by smooth muscle cells
Source: Sci Rep. 2021 Mar 15;11:5901. doi: 10.1038/s41598-021-85164-y (PMC7960740; doi:10.1038/s41598-021-85164-y)
Supplement: Supplementary file 1 — Supplementary Information [file 41598_2021_85164_MOESM1_ESM.docx]

**Mueller Polarimetric Imaging for fast macroscopic mapping of microscopic collagen matrix remodeling by smooth muscle cells**

Olga Chashchina^1^, Hachem Mezouar^2^, Jérémy Vizet^2^, Clothilde Raoux^3^, Junha Park^2^, Clara Ramón-Lozano^1^, Marie-Claire Schanne-Klein^3^, Abdul I. Barakat^1^, Angelo Pierangelo^2,*^

^1^, Hydrodynamics Laboratory (CNRS UMR7646), Ecole Polytechnique, IP Paris, France

^2^, LPICM (CNRS UMR 7647), Ecole polytechnique, IP Paris, France

^3^, LOB, CNRS, Inserm, Ecole polytechnique, IP Paris, France

*, corresponding author, *angelo.pierangelo@polytechnique.edu*

# Supplementary figures

| 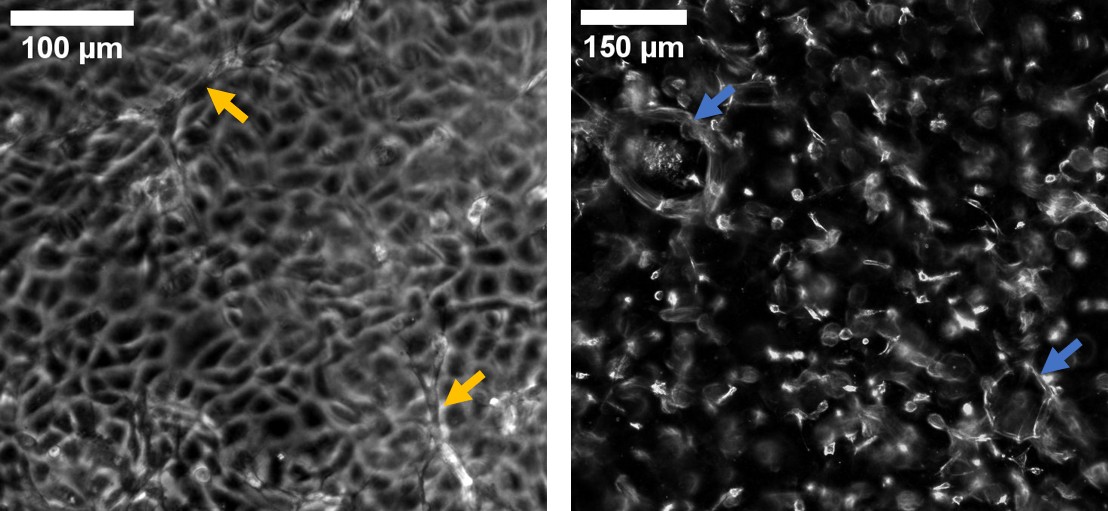 |
| --- |
| **Figure S1:** Brightfield images of: **Left**, A monolayer of ECs on top of the collagen hydrogel. Orange arrows indicate SMCs close to the surface beneath the ECs. **Right**, SMCs (bright shapes; cells are not necessarily positioned in the focal plane due to the 3D structure of the sample) in the gel. Blue arrows indicate the traces of trapped air bubbles inside the gel generated during its preparation.   \| 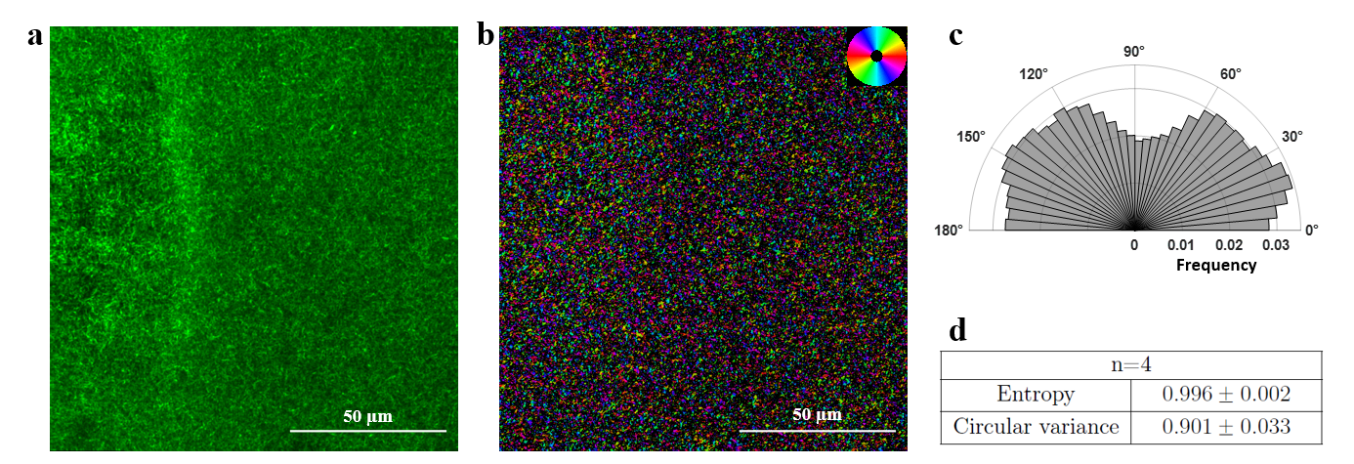 \| \| --- \| \| **Figure S2:** SHG imaging of collagen I hydrogels (C). **a)** SHG image, **b)** pSHG image and **c)** the corresponding orientation distribution of a cell-free collagen hydrogel (C). The collagen orientation in the pSHG image is color-coded according to the circular scale at the upper right corner. **d)** Mean values of entropy and circular variance within a 95% confidence interval calculated from 4 different C samples. These values are close to 1, which shows that there is no preferred orientation of collagen fibrils in the hydrogels. \| |
|  |
| **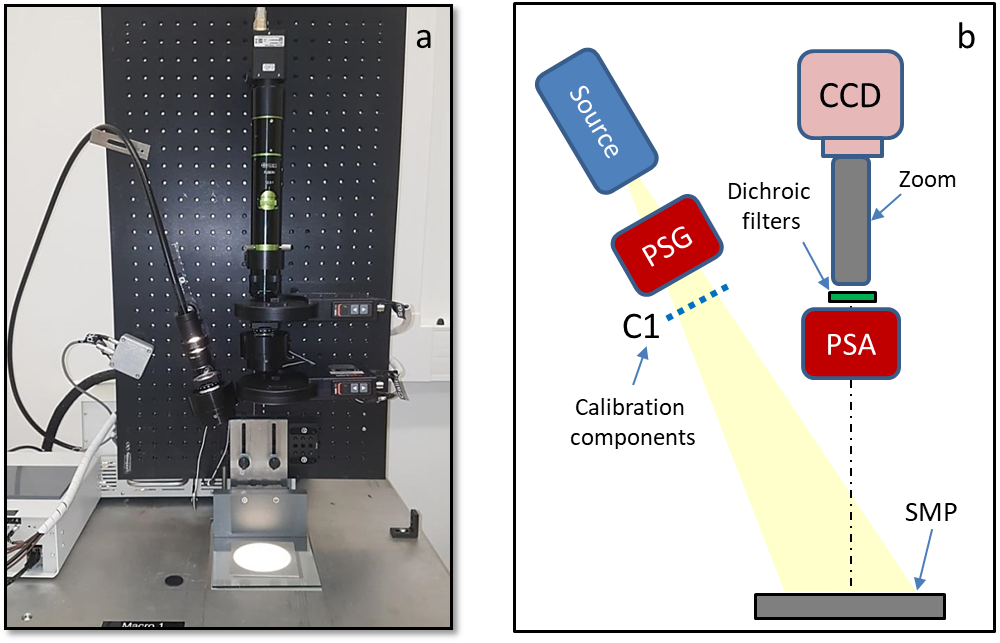**  **Figure S3: Mueller Polarimetric Imager. a)** Photograph of the optical setup. **b)** Schematic drawing of the used Mueller polarimeter. The polarimeter consists of an imaging setup with two integrated active polarimetric elements: a Polarisation State Generator (PSG) and a Polarisation State Analyser (PSA). C1 indicates the position where the optical elements used for calibration are inserted, CCD denotes charge-coupled device camera, and SMP denotes sand-blasted metallic plate. |

| 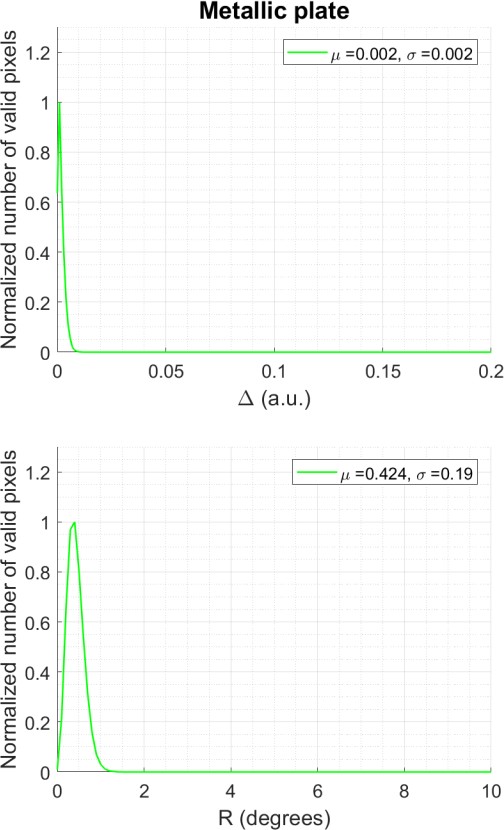 |
| --- |
| **Figure S4:** Depolarization **(top)** and retardance **(bottom)** histograms for the metallic plate representative of the ambient noise level. *µ* corresponds to the sample’s mean value. *σ* corresponds to the standard deviation of the mean. |
| 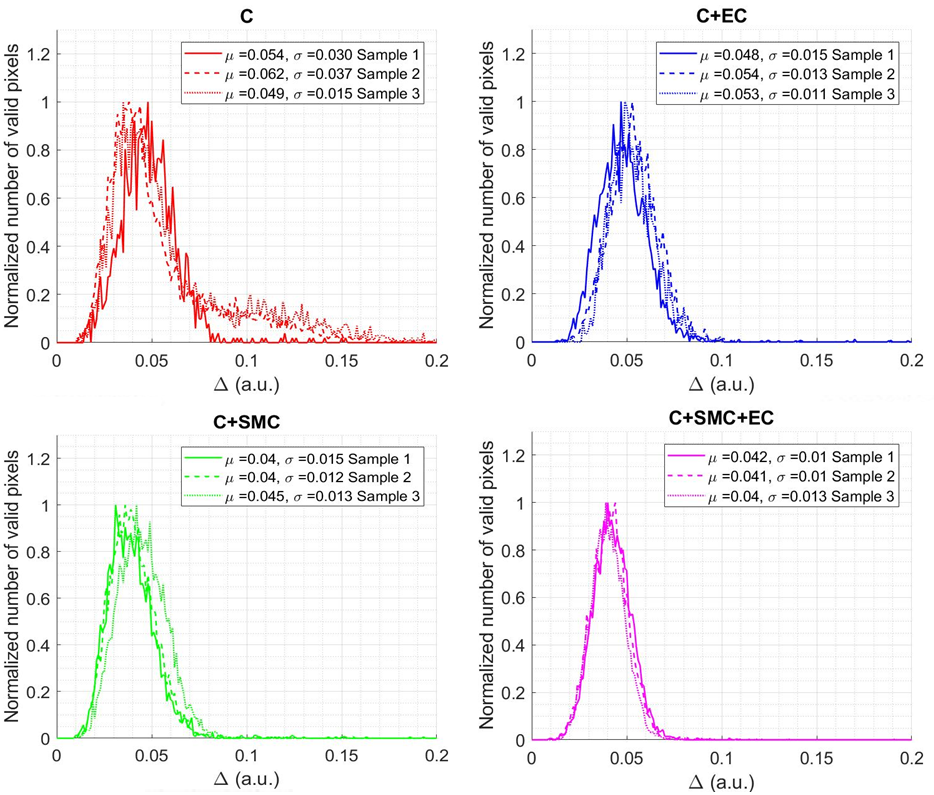 |
| **Figure S5:** Depolarization histograms for all analyzed samples based on the measurements within the yellow zone shown in **Fig. 3a**. *µ* corresponds to the mean depolarization value of an individual sample. *σ* corresponds to the standard deviation of the mean. Three different samples were analyzed for each condition. |
|  |
| 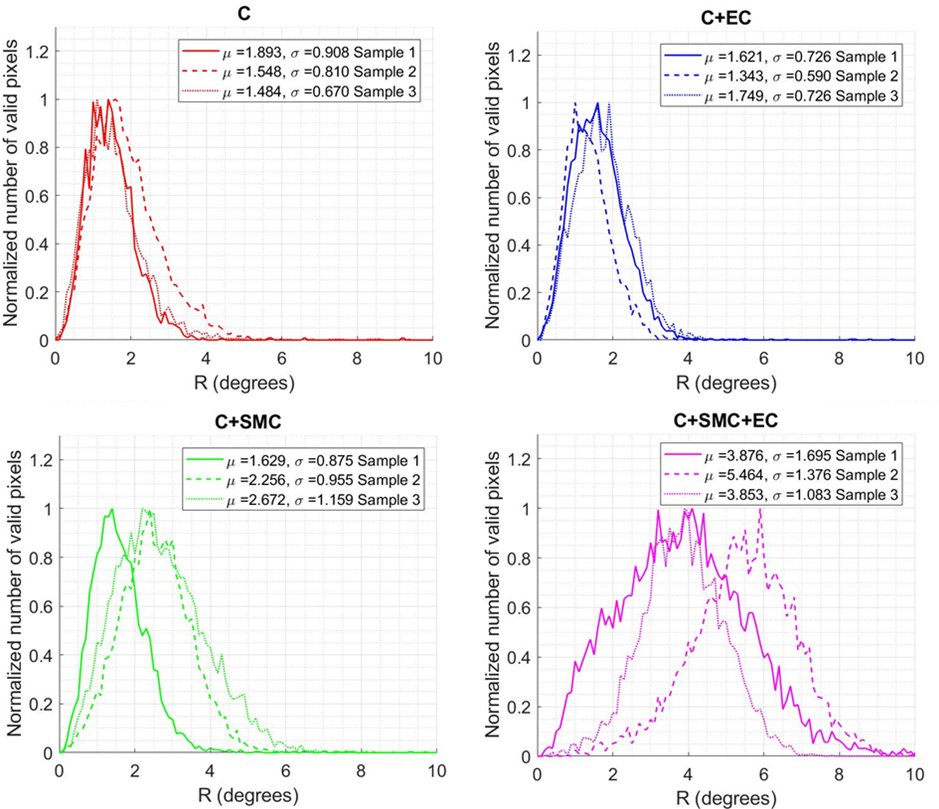  **Figure S6:** Retardance histograms for all analyzed samples based on the measurements within the yellow zone shown in **Fig. 3a**. *µ* corresponds to the mean retardance value of an individual sample. *σ* corresponds to the standard deviation of the mean. Three different samples were analyzed for each condition. |
